# Next-generation sequencing technologies and applications for human genetic history and forensics

**DOI:** 10.1186/2041-2223-2-23

**Published:** 2011-11-24

**Authors:** Eva C Berglund, Anna Kiialainen, Ann-Christine Syvänen

**Affiliations:** 1 Department of Medical Sciences, Molecular Medicine and Science for Life Laboratory, Uppsala University, 751 85 Uppsala, Sweden

## Abstract

Rapid advances in the development of sequencing technologies in recent years have enabled an increasing number of applications in biology and medicine. Here, we review key technical aspects of the preparation of DNA templates for sequencing, the biochemical reaction principles and assay formats underlying next-generation sequencing systems, methods for imaging and base calling, quality control, and bioinformatic approaches for sequence alignment, variant calling and assembly. We also discuss some of the most important advances that the new sequencing technologies have brought to the fields of human population genetics, human genetic history and forensic genetics.

## Background

Determining the DNA sequence is the most comprehensive way of obtaining information about the genome of any living organism. For decades, Sanger sequencing [1] using fluorescently labeled terminating nucleotides and electrophoresis has been the gold standard sequencing technology. Sanger sequencing made an early impact in the field of microbial genomics, with the first complete bacterial genome, *Haemophilus influenzae*, sequenced in 1995 [2]. Multicenter collaborations using numerous sequencing instruments and automated sample preparation also made it possible to use Sanger sequencing in the human genome project, which took more than 10 years and US$2.7 billion to complete [3,4].

In recent years, we have witnessed a rapid development of a new generation of DNA sequencing systems followed by a multitude of novel applications in biology and medicine. The major advantage of the new 'second-generation' or 'massively parallel' sequencing technologies, compared to Sanger sequencing, is their considerably higher throughput and thereby lower cost per sequenced base. On a second-generation sequencing (SGS) machine several human genomes can be sequenced in a single run in a matter of days. Here, we review recent technological advances of SGS technologies and discuss the bioinformatic and computational implications of the sequencing revolution. Finally we highlight some applications of SGS technology with a focus on human population genetics and genetic history, and genetic forensics.

### Second-generation sequencing technologies

**Figure 1:**
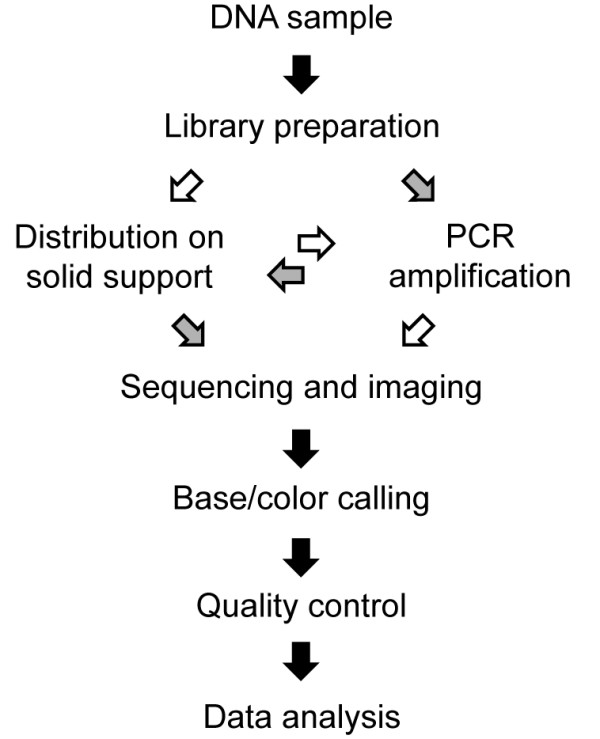
**Steps of a sequencing experiment**. Black arrows indicate steps that are common for all second-generation sequencing (SGS) technologies, white arrows refer to the Illumina systems, and grey arrows refer to the Roche 454 and SOLiD systems.

There are three major SGS systems that are routinely used in many laboratories today. The first system to become commercially available was the Genome Sequencer from 454 Life Sciences (Branford, CT, USA) (later acquired by Roche [5]) in 2005, which was also the first SGS technology to sequence a complete human genome, that of Dr. James D. Watson [6]. The Genome Analyzer, first conceived by Solexa and later further developed by Illumina (San Diego, CA, USA) [7] was launched in 2006, and the SOLiD system from Applied Biosystems [8] (now part of Life Technologies (Carlsbad, CA, USA)) in 2007. The key steps of a sequencing project are the same for all of these technologies: preparation and amplification of template DNA, distribution of templates on a solid support, sequencing and imaging, base calling, quality control and data analysis (Figure 1).

In terms of applications, there are two major types of projects, *de novo *sequencing and resequencing. In a *de novo *sequencing project, the genome of an organism is sequenced for the first time. In contrast, in resequencing applications, the genome or parts of it are sequenced of a species where a reference sequence is already available. This difference affects both the selection of sequencing strategy and the data analysis (further discussed below). In human forensics and population genetics the resequencing approach is used, but in microbial forensics both *de novo *sequencing and resequencing of microbial genomes may be required.

Two common measures of the amount of sequence data generated in a project are the sequencing depth and breadth. Sequencing depth, or coverage, is the average number of times each base in the genome is sequenced. For example, to sequence a 3 Gb human genome to 30 × coverage, 90 Gb of sequence data is needed. The coverage will be uneven over the genome however, and sequencing breadth, sometimes also referred to as genome coverage, is the percentage of the genome that is covered by sequence reads.

### DNA samples for sequencing

High-quality DNA in sufficient quantity is the basis for any successful sequencing experiment. For most sequencing applications, 1 to 5 μg of purified DNA is needed, an amount that may not always be available. Whole genome amplification (WGA) has frequently been used to increase the amount of DNA for genotyping [9] and can be applied also in combination with SGS. Several microbial genomes have been sequenced using SGS after WGA (for example, the genome of uncultured bacterial symbionts of termites isolated from a single host cell [10]). WGA was also recently used to amplify DNA from single cells from primary breast tumors, and although sequence data was retrieved from only 6% of the genome of each cell, this genomic representation was enough to identify subpopulations of cancer cells by copy number variations [11].

SGS can also be used to detect rare and unknown variants in genomic regions of interest in a cost-efficient way, and in a larger number of samples than by whole genome sequencing. Another advantage of targeted sequencing is the reduced issue of sequence reads aligning to multiple locations in the genome. The most commonly used methods for enrichment of genomic regions for sequencing are either based on hybridization to biotinylated probes in solution or probes immobilized on microarrays, or on multiplexed amplification by PCR (reviewed in [12]). The recently developed selector probe technology, which is based on rolling circle amplification, provides efficient and highly multiplexed enrichment of small regions totaling up to 1 Mb in size is particularly useful for ultra-deep sequencing at low cost and high specificity [13]. Sequencing of human exomes enriched by hybridization-based capture in solution is becoming widely used, and has proven to be particularly successful for identification of mutated genes underlying monogenic disorders (see, for example, [14-17]). The methods for hybridization-based capture are also applicable to custom-selected genomic regions of interest [18].

### Preparation of sequencing libraries

The DNA samples to be sequenced are first converted into one of two main types of sequencing libraries, fragment libraries or mate-pair libraries. The first step in the preparation of a sequencing library is to fragment the DNA sample, usually using sonication or nebulization. For preparation of fragment libraries, sequencing adapters are ligated to both ends of the DNA fragments, followed by PCR amplification using primers complementary to the adapters.

In the Illumina SGS technology, adapter-ligated DNA fragments are amplified directly in the flow cell subsequently used for sequencing. Each flow cell has eight channels (lanes) coated with oligonucleotides that are complementary to the adapters. The adapter-ligated DNA fragments are hybridized to the flow cell, in which they are distributed randomly and amplified by a process called bridge amplification. After amplification, DNA molecules are linearized to form clusters, each of which consists of about 1,000 copies of the original DNA molecule at that position.

In the 454 and SOLiD technologies, adapter-ligated fragments are hybridized to beads coated with an oligonucleotide that is complementary to one of the adapters for amplification in a water-in-oil emulsion PCR. Each water droplet constitutes a microreactor containing the PCR reagents and optimally a single bead with a single immobilized DNA fragment. Thus, multiple PCRs can be performed in parallel in a single tube. After breaking the emulsion, the beads, which are now coated with thousands or millions of copies of the original DNA molecule, are loaded onto the solid support for sequencing. In the 454 system, the solid support is called PicoTiterPlate and consists of wells that can fit a single DNA-coated bead each. SOLiD uses a glass slide to which the beads are distributed randomly.

The amplified fragments are then sequenced either from one end (single-end) or from both ends (paired-end). Paired reads allow more accurate alignment to a reference genome, and are also very useful to resolve repeats and improve assembly in *de novo *sequencing projects. The Illumina system generates sequence reads of the same length from both ends, whereas the second read from SOLiD is shorter (Table 1). The 454 system currently does not support paired-end sequencing of fragment libraries.

**Table 1:** Characteristics of second-generation and third-generation sequencing instruments

Instrument	Read length (nucleotides)	No. of reads^a^	Output (Gb)^a^	No. of samples^a, b^	Runtime	Advantages	Disadvantages
Roche 454 GS FLX+	700^c^	1 × 10^6^	0.7	192^d^	23 h	Long reads, short run time	Homopolymer errors, expensive
Illumina HiSeq2000	100^e^	3 × 10^9^	600	384	11 days^f^	High yield	No. of index tags limiting
Life Technologies SOLiD 5500xl	75^g^	1.5 × 10^9^	180	1,152	14 days^f^	Inherent error correction	Short reads^g^
Roche 454 GS Junior	400^c^	1 × 10^5^	0.035	132	9 h	Long reads	Homopolymer errors, expensive
Illumina MiSeq	150	5 × 10^6^	1.5	96	27 h	Short run time, ease of use	Expensive per base
Ion Torrent PGM Ion 316 chip	> 100^h^	1 × 10^6^	0.1	16	2 h	Short run time, low reagent cost	Not well evaluated
Helicos BioSciences HeliScope	35^h^	1 × 10^9^	35	4,800	8 days	SMS, sequences RNA	Short reads, high error rate
Pacific Biosciences PacBio RS	> 1,000^h^	1 × 10^5^	0.1	1	90 min	SMS, long reads, short run time	High error rate, low yield

Most of this information is subject to rapid change, and the aim of this table is not to present absolute numbers but to provide a general comparison between different sequencing systems.

^a^Numbers calculated for two flow cells on HiSeq2000 and SOLiD 5500xl.

^b^Calculated as no. of index tags (provided by the sequencing company) × no. of divisions on solid support.

^c^Average for single-end sequencing, paired-end reads are shorter.

^d^No. of reads decreases when the PicoTiterPlate is divided.

^e^36 nucleotides for mate-pair reads.

^f^Run time depends on the read length, and on whether one or two flow cells are used.

^g^Second read in paired-end sequencing is limited to 35 nucleotides, and mate pair reads to 60 nucleotides.

^h^Average.

SMS = single molecule sequencing.

**Figure 2:**
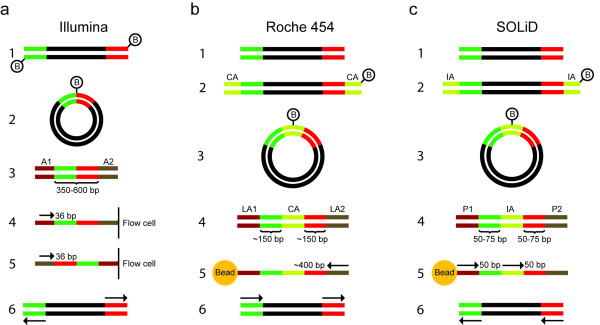
**Principles for construction of mate-pair sequencing libraries**. **(a) **Preparation of Illumina mate-pair libraries. Fragments are end-repaired using biotinylated nucleotides (1). After circularization, the two fragment ends (green and red) become located adjacent to each other (2). The circularized DNA is fragmented, and biotinylated fragments are purified by affinity capture. Sequencing adapters (A1 and A2) are ligated to the ends of the captured fragments (3), and the fragments are hybridized to a flow cell, in which they are bridge amplified. The first sequence read is obtained with adapter A2 bound to the flow cell (4). The complementary strand is synthesized and linearized with adapter A1 bound to the flow cell, and the second sequence read is obtained (5). The two sequence reads (arrows) will be directed outwards from the original fragment (6). **(b) **Preparation of Roche 454 paired-end libraries (these are called paired-end, but are based on the same principles as the mate-pair libraries in the other technologies). Original fragments (1) are end-repaired with unlabeled nucleotides, and biotin-labeled circularization adapters (CA) are ligated to the fragment ends (2). After circularization (3), fragmentation and affinity purification, library adaptors (LA1 and LA2) are ligated to the new fragment ends (4) and the fragments are amplified on beads by emulsion PCR. One single sequence read that covers the two original ends and the internal adapter is generated (5). Adapter sequence is removed *in silico*, and the sequence is split into two reads, which both have the same orientation (6). **(c) **Preparation of SOLiD mate-pair libraries. Steps 1 to 4 are analogous with preparation of Roche 454 paired-end libraries, with a biotin-labeled internal adapter (IA) and two sequencing adapters (P1 and P2). Sequencing is performed with two different primers, complementary to the P1 adapter and internal adapter, respectively (5). The resulting reads will have the same orientation (6).

Mate-pair libraries are constructed by circularizing fragmented DNA, thereby bringing the two ends of the original DNA fragment adjacent to each other (Figure 2). After fragmentation of the circular DNA, the fragment containing the ends of the original linear DNA is selected using biotin capture. Sequencing both ends of the selected fragment will yield reads that are separated by the distance of the original fragment. In order to avoid chimeric sequence reads that span over both original fragment ends, the 454 and SOLiD systems include an internal adapter. In the Illumina mate-pair preparation, no internal adapter is used, and to minimize the risk of sequencing over the original junction the recommended read length is limited to 36 nucleotides. Mate-pair libraries allow larger insert sizes (2 to 20 kb) than paired-end sequencing of fragment libraries. Drawbacks of mate-pair sequencing are that the laboratory protocols are more complicated and that a substantially larger amount of DNA (5 to 120 μg) is required. In contrast to paired-end reads, which are oriented towards each other, mate-pair reads are either both oriented outwards from the original fragment or both have the same orientation (Figure 2), which needs to be accounted for in the data analysis. Large inserts are especially valuable in *de novo *sequencing projects, where they can substantially improve scaffolding(ordering of assembled contigs). Mate-pair sequencing is not used as frequently in resequencing projects, where DNA resources are often limited and the analysis is mainly based on alignment to a reference genome.

Introducing an additional index tag (barcoding) to each DNA fragment makes it possible to sequence pooled samples that can be distinguished *in silico *after sequencing. Multiplexing is useful in applications where a relatively small amount of data is needed from each sample, such as sequencing of small genomes or enriched regions of large genomes (see, for example, [19,20]). As the capacity of the sequencing instruments has increased, multiplex sequencing of indexed samples has become more and more important to minimize sequencing costs. Indexing also decreases the risk of sample mix-ups and contaminations during library preparation. Currently, Illumina provides 24 different index tags, Life Technologies 96 and Roche 12 (Table 1). Additional index tags for Illumina (48 tags in total) and 454 (120 additional tags) can be purchased from other companies. It is also possible to use custom-designed index tags [21,22].

### Sequencing and imaging principles

#### Sequencing-by-synthesis

**Figure 3:**
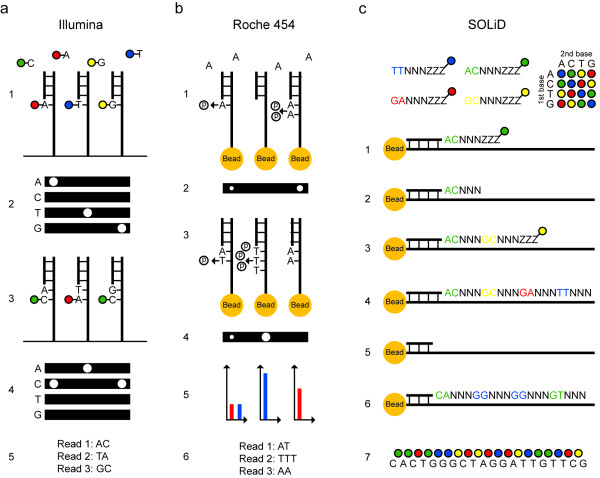
**Principles for sequencing and imaging**. **(a) **Illumina sequencing of three template molecules. All four nucleotides, carrying terminating moieties and unique fluorescent labels, and DNA polymerase are added, and one complementary nucleotide becomes incorporated at each template molecule (1). After washing, fluorescence is registered at four wavelengths (2). Fluorescent dyes and terminating groups are cleaved off. A new set of nucleotides is added (3), and imaged (4). Sequence reads of equal length are obtained (5). **(b) **454 sequencing of three template molecules. One type of natural non-terminating deoxynucleotides and DNA polymerase are added and a pyrophosphate molecule is released at each nucleotide incorporation (1). Pyrophosphate is converted into light using sulfurylase and luciferase, and the light intensity is measured in each well (2). Free deoxynucleotides are destroyed with apyrase before adding the next type of deoxynucleotide (3) and imaging (4). Light signals are converted to flowgrams with higher signal intensity bars in homopolymer regions (5). Sequence reads that may differ in length are obtained (6). **(c) **SOLiD sequencing of one template molecule. A sequencing primer, DNA ligase and 1,024 unique probes, which are fluorescently labeled according to their first two bases, are added, and the complementary probe is ligated to the template (1). After washing, fluorescence is registered at four wavelengths. The three universal bases and the fluorophor are cleaved off (2). Addition of a new probe set is repeated for the desired number of cycles (3,4). The newly built strand is melted off. A new sequencing primer is added, which anneals one base off from the first primer and therefore interrogates different positions (5). Sequencing is repeated for the desired number of cycles (6). Additional primers are added, until each base is sequenced twice. The colors from all sequencing rounds are merged and can be converted to nucleotides (7).

The Illumina and 454 technologies are based on sequencing-by-synthesis. A DNA polymerase is used to extend a sequencing primer by incorporating nucleotides that form a growing sequence complementary to the template DNA. In the Illumina system, fluorescent reversibly terminating nucleotides are used. All four nucleotides are added at the same time, each with a unique fluorescent label, which allows incorporation of one base per cycle into each template molecule [23] (Figure 3a). After incorporation and fluorescence registration at four wavelengths, the terminating and fluorescent moieties are removed from the nucleotides to allow the next sequencing cycle. In 2010, Illumina released the HiSeq2000 instrument, which uses the same chemistry as the original Genome Analyzer instrument, but has improved imaging optics and can process two flow cells in parallel. The HiSeq2000 system has the highest throughput of all currently available SGS instruments, with around 600 Gb sequence produced per run (Table 1). Examples of what can be achieved with the current capacity of HiSeq2000 are shown in Table 2. Sequencing errors are primarily substitution errors and occur more frequently in the distal bases of a read.

**Table 2:** Capacity of the HiSeq2000 instrument from Illumina

Target region	Coverage	Samples per run
Human genome (3 Gb)	40 ×	5
Human exome (30 Mb)	100 ×	200
*Escherichia coli *genome (6 Mb)	200 ×	500
Ten large genes (1 Mb)	100 ×	6,000

In the 454 sequencing-by-synthesis reaction, natural non-terminating deoxynucleotides are added to the system sequentially (Figure 3b). In homopolymeric regions several bases will thus become incorporated in the same step. The 454 technology is based on the pyrosequencing principle [24], where pyrophosphate is released as a consequence of nucleotide incorporation and converted into ATP by sulfurylase. ATP is then used as a substrate for the production of light by luciferase, and the emission of light is registered by a charge-coupled device (CCD) camera. The major advantage of the 454 technology is the long read length. The misincorporation rate for the natural deoxynucleotides is low, resulting in low levels of nucleotide substitution errors. Insertion-deletion errors are frequent in homopolymeric regions, however, due to the non-linear light response when several nucleotides are incorporated simultaneously to the same molecule. Compared to the Illumina and SOLiD systems, the 454 technology is more expensive per base due to the lower capacity and the higher reagent cost associated with the multiple enzymes required. Thus, the 454 technology is mainly used in applications where long reads are desired, such as *de novo *sequencing projects, where the read length is the most important factor determining the quality of the assembly, and in metagenomics, where the sample contains a mix of different organisms.

#### Sequencing-by-ligation

The SOLiD technology is based on sequencing-by-ligation, where a DNA ligase is used to add probes to a growing oligonucleotide chain [25] (Figure 3c). The probes consist of eight bases, five that are specific and complementary to the template and three that are universal and support hybridization to the DNA template. In the sequencing reaction, probes containing all possible combinations of the first five nucleotides are added. The probe that matches the template perfectly becomes hybridized and ligated to the sequencing primer or previous probe. The probes are fluorescently labeled according to the first two bases using a scheme for two-base encoding with four fluorophores. After imaging, the fluorescent label and the three universal bases are cleaved off, and a new set of probes is added. After the first round of sequencing, the newly built DNA strand is melted off, a new sequencing primer which starts one base off from the first primer binding site is hybridized, and the sequencing reaction is repeated now interrogating different positions. This process is repeated several times with different primers, so that all bases in the template become sequenced twice. Since each base is sequenced twice in the SOLiD system, most sequencing errors can be corrected during alignment, resulting in a low error rate of mapped data. It is possible to use different chemistries and read lengths in different lanes. The current read length is 75 nucleotides for fragment libraries, and the total yield per run (two glass slides) is around 180 Gb. Data analysis has traditionally been performed in color space, however, with the recent upgrade to the 5500xl system, it is now also possible to get error corrected reads in base space.

Sequencing-by-ligation is also used by Complete Genomics (Mountain View, CA, USA) [26], a company that sequences human genomes as a service. In their technology, the template DNA is first inserted into a single-stranded DNA circle, which is then copied several times to make up DNA nanoballs. The nanoballs are attached to arrays and sequenced by ligation reactions, which use multiple priming sites [27]. The current capacity of Complete Genomics is more than 600 genomes per month, and they are driving down the price of whole-genome sequencing.

#### Base calling and quality control

Intensities of light signals from the sequencing reactions are converted to bases (Illumina and 454 systems) or colors (SOLiD system). In addition to sequence data, base calling produces quality scores for each base, which are estimates of the probability of the call being erroneous. After base calling, reads with indications of mixed signals or other errors are filtered out. To facilitate troubleshooting and discrimination between problems caused by instrument/reagent factors and sample factors, the Illumina and 454 platforms include standardized control DNA in each run.

In the Illumina system, the clusters are identified during the first four cycles of sequencing. Intensities are registered for each cluster in every cycle and converted to nucleotide sequence. If the initial recognition of clusters was not perfect, some clusters may contain more than one original template molecule. It is also possible that some clusters contain many molecules that have incorporated fewer (phasing) or more (prephasing) nucleotides than the number of cycles. Such clusters are filtered out by a so-called chastity filter, which is based on the ratio of signal intensities of the bases with the strongest and the second strongest intensity. Control DNA from the phage phiX is sequenced in each flow cell. In the analysis pipeline, phiX reads are identified by comparison to the phiX genome and the error rate is determined and used as a measure of the quality of the run.

During QC filtering of data from the 454 system, possible polyclonal beads and beads with no template are identified based on the number of positive and negative flows. In addition, reads that do not start with a specific key sequence, which is part of the adapter, and reads that have a high number of off-peak signal intensities (indicative of homopolymer errors) are filtered out. Sequence reads are also trimmed from the 3' end to remove adapter sequence and bases of low quality, arising from phasing/prephasing issues and loss of signal intensity. Beads with control DNA, labeled with a different key sequence, are included in each run. With the aid of the key sequence, these sequence reads are identified and aligned to a reference sequence, and the percentage of reads that match with 95, 98 and 100% similarity is reported.

After color calling in the SOLiD system, possible polyclonal reads and reads with color combinations that do not make sense according to the two-base encoding scheme are filtered out. No control DNA is used, but the quality of a run is assessed from the color intensity distribution.

Despite the standard QC steps, not all obtained data will be of high quality. To recognize potential problems and biases it is useful to apply additional quality control measures. A good resource for assessing the quality of sequence data is the FastQC software [28], which reports, for example, distributions of base qualities, GC content, redundancy and over-representation of adapter or primer sequence.

### Trends and upcoming technologies

#### Low-capacity sequencing systems

While the sequencing companies compete to increase throughput, they have also launched systems with lower capacity. The reason for this trend is that the high capacity of the original systems is not always required, and the current multiplexing possibilities do not match their throughput, which results in a much higher coverage (and cost) than needed for many applications.

Roche 454 Technologies was first to launch the Genome Sequencer Junior system in 2010 and Illumina launched the MiSeq system in 2011. The capacity of these systems is 35 Mb and 1.5 Gb per run, respectively. Life Technologies recently acquired the company Ion Torrent, whose technology has a concept similar to that of the 454 system. However, detection is based on pH changes caused by release of electrons upon nucleotide incorporation rather than pyrophosphate release. Since no enzymes are used for detection, the reagent cost for this instrument is low.

Compared to the high-capacity instruments, the cost per base is high for the smaller machines, but they are suitable for sequencing small genomes, amplicons or DNA enriched by targeted capture. Since the run time for these systems is short, they are also useful for technology development runs and to optimize reaction conditions for a larger run. These machines have been referred to as 'personal sequencers', meaning that they are easily obtainable also by laboratories with smaller resources that want to have rapid access to a sequencing instrument, but need relatively small amounts of data.

#### Single molecule sequencing

In single molecule sequencing, sometimes also referred to as third-generation sequencing, no amplification of the template molecules is performed prior to sequencing. These technologies provide improved quantitative accuracy by eliminating the risk of biases introduced during preparation of sequencing libraries. Single molecule sequencing also allows direct sequencing of RNA molecules, detection of chemically modified bases such as DNA methylation, and increased read lengths. Longer reads will be useful in *de novo *sequencing projects and open up perspectives for experimental phasing (determination of which variant alleles are on the same chromosome), in contrast to statistical phasing that is used today.

In the Heliscope Single Molecule Sequencer system from Helicos Biosciences (Cambridge, MA, USA) [29] single stranded poly(dA)-tailed templates are attached to poly(dT) oligonucleotide primers that are anchored on a flow cell. In each sequencing cycle one type of reversibly terminating fluorescently labeled nucleotides are added and incorporated by a polymerase, the slide is washed and imaged, and the dye labels are cleaved off [30,31]. This technology generates around 35 Gb per run, and the read length is 35 nucleotides on average (Table 1).

Pacific Biosciences (Menlo Park, CA, USA) [32] has developed a system called single molecule real time (SMRT) sequencing, which uses a DNA polymerase anchored on a glass surface and nucleotides with phospholinked fluorescent labels that are cleaved off when the nucleotides are incorporated. The sequencing reaction takes place on zero-mode waveguide nanostructure arrays [33]. The incorporation of the fluorescently labeled nucleotides is monitored in real time, which results in very short run times. This system has read lengths over a thousand bases, but error rates are high and throughput is currently limited to 0.1 Gb per run (Table 1). The Pacific Biosciences system was successfully used for rapid analysis of the *Cholera *strains in the outbreak in Haiti in 2010 [34].

Several new technologies for single molecule sequencing are under development. Nanopore sequencing technologies (Oxford Nanopore (Oxford, UK) [35], NABsys (Providence, RI, USA) [36]) are based on detecting natural electric or chemical differences between nucleotides, and do not require labeling of DNA. The Starlight system (Life Technologies) is a real-time technology that uses a quantum-dot-labeled polymerase and distinctly labeled fluorescent nucleotides. The reaction and detection principles underlying the third-generation sequencing systems are demanding, and today it is not obvious which of these systems that will reach the capacity and accuracy required for practical 'real life' applications.

### Bioinformatic analysis of sequence data

#### Alignment

**Figure 4:**
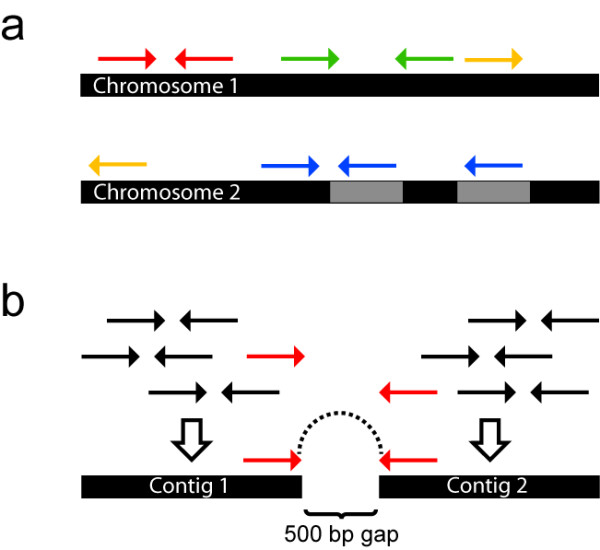
**Principles of reference alignment and *de novo *assembly**. **(a) **Alignment of paired-end reads to two chromosomes of a reference genome. Arrows with the same color indicate reads that belong to the same pair. Red arrows illustrate a normal pair, aligning with the expected orientation and distance. Green arrows illustrate a pair that aligns at a larger distance than expected due to a potential deletion in the sequenced genome. Orange arrows illustrate a pair that aligns to different chromosomes indicating a potential rearrangement in the sequenced genome. Blue arrows illustrate how paired-end reads can guide alignment if one of the reads aligns in a repeated (grey) region. **(b) ***De novo *assembly of paired-end reads without the guidance of a reference. Overlapping reads (arrows) are assembled into clusters, and the consensus sequence of each cluster is called a contig. Reads of the same pair that belong to different contigs (red arrows) can help to order contigs into scaffolds. Because the average size of the original fragments is known, the size of the gap between the contigs can be estimated.

For resequencing applications, where a reference sequence is already available, the first step of the analysis is usually to align the sequence reads to the reference genome (Figure 4a). In recent years, several novel alignment programs have been developed that are adapted to the shorter read lengths, different error distributions and larger data amounts obtained by SGS technologies (reviewed in [37]). These programs have different properties in terms of, for example, their ability to perform gapped alignment, how base qualities are used during alignment and how reads aligning to repeated regions are treated. Some aligners can handle data from any sequencing platform, whereas others are specific to one platform.

To save computational time, the alignment is usually performed in two steps: (1) a limited number of candidate positions are identified by fast heuristic approaches, and (2) candidate positions are evaluated by more accurate methods, such as the Smith-Waterman algorithm [37]. The first step of the alignment is typically based either on hash tables or on the FM index (named for its creators, Paolo Ferragina and Giovanni Manzini). Usually, the algorithms do not map the entire read at once, but start with a seed sequence consisting of the first 20 to 40 bases. The seed is made up of consecutive bases, or allows for mismatches at certain positions (spaced seeds). To improve sensitivity, several different spaced seeds can be used. To improve speed, some programs discard reads with too many candidate positions.

Regardless of which aligner is selected, there is always a trade off between speed and sensitivity. As the amount of data has increased, algorithms based on the FM index (for example, Bowtie [38] and Burrows-Wheeler Aligner (BWA) [39]) have become increasingly popular. This index is based on the Burrows Wheeler transform, which was originally used in algorithms for data compression and is efficient for large amounts of data. The expected divergence between the reference sequence and the reads also influences the efficiency of alignment programs. While the BWA loses rapidly in speed when the difference between the reads and the reference is more than 4%, the use of several spaced seeds in BFAST [40] makes it highly tolerant to divergent reads.

#### Variant calling

There are multiple programs for identifying single nucleotide polymorphisms (SNPs), copy number variations (CNVs) and structural variants between sequence reads and a reference genome (reviewed in [41]). These programs aim to distinguish authentic variants from sequencing errors and incorrect alignments by evaluating different parameters, such as base qualities, coverage and the number of reads supporting the variant. Different combinations of aligners and variant callers typically yield different results, and there is no consensus in the scientific community on which algorithms are most appropriate to use. An approach that was used in the 1000 Genomes Project is to run several variant callers, and report the variants detected by at least two different methods.

Many variant callers tend to overcall, and it is often necessary to filter the detected variants. It is also possible to improve the results by local realignment around candidate variants and recalibration of quality scores before variant calling, using the software suite GATK [42]. During realignment, all reads around potential indels (insertions and deletions) are extracted and subsequently aligned again in a multiple alignment with exact methods. This can remove many false positive variants, since alignments are especially error prone around indels. During recalibration, different types of information, such as base quality scores, position in the read and dinucleotide content at positions present in dbSNP is used to improve the base quality scores.

#### *De novo *assembly

In a *de novo *sequencing project, sequence reads are aligned to each other without the guidance of a reference sequence, with the aim to assemble as long contiguous sequences (contigs) as possible (Figure 4b). This process is computationally much more challenging than alignment to a reference sequence. For example, running an assembly of a human genome requires around 150 GB of RAM using SOAPdenovo [43] and around 512 GB of RAM using Allpaths-LG [44]. In contrast, aligning paired-end sequences to a reference using BWA only requires 3.5 GB of RAM, and can be done on a standard desktop computer for a limited number of samples. Long reads and paired-end or mate-pair data help to resolve repeated regions, and will greatly improve the assembly. Since throughput, read length and error distributions differ between the platforms, a combination of data can often be useful for *de novo *sequencing, such as 454 data to obtain long reads and Illumina data to increase the coverage and correct homopolymer errors. As of October 2011, the number of published genomes sequenced with SGS is approximately the same as those sequenced with Sanger sequencing according to the Genomes OnLine Database (GOLD) [45]. However, among approximately 1,000 listed ongoing projects, Sanger sequencing is only used in 10%, with most of the genomes being sequenced with Illumina (49%) or 454 (40%). 454 sequencing is more frequently used for bacterial and archaeal genomes than for eukaryotes.

Algorithms developed for *de novo *assembly of short reads are based on the de Bruijn graph rather than the overlap graph, which is used in most assemblers of Sanger sequencing data. In the de Bruijn graph, reads are decomposed into shorter pieces of length k (k-mers), and overlaps between the k-mers are identified [46]. Memory and time requirements increase rapidly with the number of unique k-mers, which in turn is heavily influenced by sequencing errors. Therefore, some programs attempt to correct sequencing errors by substituting k-mers with very low incidence with the most similar high-incidence k-mer. Despite novel algorithms, *de novo *assembly of large genomes remains very difficult, and it is also hard to evaluate the quality of an assembly. As an attempt to advance this field, the Genome Center at University of California Davis and researchers at University of California Santa Cruz recently launched a genome assembly competition called The Assemblathon, based on both real and simulated data [47].

#### Computer resources

The increasing output of the new sequencing technologies continually raises the requirements for computer resources and bioinformatic competence to handle the data [48]. Furthermore, the rate of generation of sequence data increases much faster than the capacity of storing data, suggesting that storage might become a major bottleneck. To circumvent this, there are ongoing efforts to develop a more efficient file format, the CRAM format, which is based on storing the differences compared to a reference rather than the reads themselves [49]. The CRAM format requires five times less storage space than the BAM format, which is the standard today. In lack of sufficient computer power for data analysis, it is possible to analyze data using internet-based so-called cloud computing and pay for the service by the hour. Several public data sets are already available on clouds; it is for example possible to analyze the data from the 1000 Genomes Project on the Amazon Web Services Cloud [50]. The use of hardware acceleration solutions such as graphics processing units to decrease computational time is not widely employed in bioinformatics yet, but may increase in the future. An example of a program that uses this approach is Barracuda [51], a sequence aligner based on the BWA algorithm, which uses the Nvidia CUDA architecture.

A useful resource for information regarding software and analysis is SEQanswers [52], a community for discussion on new sequencing technologies. SEQanswers also lists many of the programs available for data analysis [53]. In addition to open-source software, there are also commercial software suites, like CLCbio [54], which may be more accessible for users without programming experience.

### Population genetics

Genetic information provides valuable insights on human origin and migration, and genotyping studies have revealed a distinct geographic substructure of human populations [55,56]. SGS technologies, including sequencing of complete genomes of many individuals, will allow population genetic studies at substantially increased resolution and improve detection of variation at the rare end of the scale. However, they will at the same time increase the complexity of interpretation.

The 1000 Genomes Project, which was launched in 2007 as a collaborative effort between three sequencing centers in the USA, the Sanger Institute in the UK and the Beijing Genomics Institute in China, was the first attempt to apply SGS in population genetics [57]. Analysis of the first set of data from three pilot projects revealed that each person carries an estimated 250 to 300 loss-of-function variants in annotated genes, and the *de novo *germline substitution rate was estimated to 10^-8 ^per base and generation [58]. Additionally, several hundred thousand SNPs with allele frequencies that differ between populations were identified. The enrichment of non-synonymous changes at these sites suggested an action of local adaptation [58]. Most variable sites have a low minor allele frequency, and are not shared among diverged populations [59]. The complete 1000 Genomes Project will include low-coverage genome sequencing, exome sequencing and genotyping of 2,500 individuals from 27 populations in Europe, Africa, Asia and the Americas, and will be an invaluable resource for studies of human population genetics.

Two recent studies developed new statistical methods for population genetic analysis of whole genome sequence data, to infer human demographic parameters [60-62]. The results showed that European and Chinese populations have similar population size histories, and both populations experienced a more severe bottleneck than African populations 10,000 to 60,000 years ago [60]. It was also found that the Eurasians diverged from the Africans 38,000 to 64,000 years ago and that the southern African San population diverged from other human populations even earlier, 108,000 to 157,000 years ago [61]. High-throughput sequencing of enriched mitochondrial DNA (mtDNA) of 109 randomly selected samples from three Filipino groups suggested that previous estimates of population size history of these populations, obtained from a biased sampling and Sanger sequencing of the hypervariable region 1, were erroneous, thus demonstrating the advantage of SGS methods [63]. As more and more genome sequences from diverse populations become available, it will be possible to decipher human history in much more detail than ever before.

### Human genetic history

Although population genetic studies have been powerful for studying human history, the studies have mostly concentrated on extant populations. Yet, humans have always been fascinated by extinct cultures and species. Where did they come from? What did they look like? How are they related to contemporary species? Why did they become extinct? With recent progress in retrieving and analyzing DNA from ancient archeological findings [64], the answers to these questions are now emerging.

Ancient DNA has been isolated and sequenced from several extinct animals, including mammoth, cave bear, ground sloth and moas, and has provided insights into the lifestyle of these species [65-70]. The first ancient, although relatively recent, human genome to be sequenced was from a 4,000 years old permafrost-preserved hair tuft from a Palaeo-Eskimo from Qeqertasussuk in western Greenland [71]. The sample belonged to a male of the Saqqaq culture, the first known inhabitants of Greenland. The genome was sequenced to a coverage of 20 × with 79% of the genome represented. The analysis revealed that the Saqqaq man was more closely related to populations from far eastern Siberia than to the current inhabitants of Greenland [71], suggesting that the Saqqaq culture arose from an independent migration from Siberia about 5,500 years ago. Several phenotypic characteristics of the Saqqaq man were deduced from the genome sequence, including dark and thick hair and a metabolism and body mass index adapted to a cold climate [71].

The complete mitochondrial DNA of the Neanderthals, who are the closest known relatives of present-day humans and lived in Europe and western Asia circa 400,000 to 30,000 years ago [72,73], has been sequenced from bones found in Spain, Germany, Croatia and Russia [74,75]. A draft sequence at 1.3 × coverage of the complete Neanderthal genome was generated from DNA isolated from three approximately 40,000-year-old bones found in Vindija Cave in Croatia [76]. The analysis suggested a 1% to 4% genetic contribution from Neanderthals to all present-day non-African human populations [76].

The mtDNA [77] and complete genome at 1.9 × coverage [78] of a 30,000 to 50,000 years old finger bone from an unknown hominid group, found in Denisova Cave in southern Siberia has also been sequenced. The analysis suggested that this population, called Denisovans, was a sister group to the Neanderthals. It was estimated that Denisovans contributed to approximately 5% of the genomes of the present-day population of Melanesia, the island region north and northeast of Australia.

Working with ancient DNA requires special consideration, since DNA degrades into shorter fragments with time, cytosines deaminate to uracils, and there is a high risk of contamination with modern DNA [64]. The age and preservation of the sample affects the quantity and quality of the extracted DNA. As much as 84% of the sequence reads from the permafrost-preserved Saqqaq hair were ancient, in comparison to only 1% to 5% from the ten times older Neanderthal bones, which were colonized by microbes. However, the Deninsova sample contained almost as high fraction of ancient DNA as the Saqqaq sample, with less degraded DNA than the Neanderthal sample, suggesting that many other environmental factors than age influence DNA conservation.

Analysis of sequence data from 61 non-coding autosomal regions in three historically isolated African populations indicated that admixture between modern humans and archaic hominid forms occurred also before the exit from Africa [79]. Whole genome sequence data would provide further resolution to this kind of studies.

### Forensic genetics

DNA profiling by typing short tandem repeats (STRs) is today the standard method for forensic analysis, and DNA sequencing has until now mostly been employed to the hypervariable region of mtDNA to establish the origin of and relationship between DNA samples available in low quantities. A spectacular example of forensic analysis of human mtDNA was the identification of the remains of the Russian royal family that was executed in 1918 [80]. While Sanger sequencing of the complete 16 kb mtDNA in a large amount of samples is labor intensive, sequencing of mtDNA is easily performed with the high capacity of SGS technologies. In a recent study, sequencing to very high coverage (16,700 × on average) was used to identify heteroplasmic nucleotide positions both in normal mtDNA and somatic variations in mtDNA of cancer cells [81]. The analysis also revealed differences in the mtDNA from different organs from the same individual, a finding which may have implications for forensic analysis, since an evidence sample and a reference sample can originate from different tissues [81]. It has, however, been argued that the error rate in this study was too high to meet the standards required for forensic casework [82]. The criticism was based on a comparison of the identified mutations to the mtDNA phylogeny compiled in the PhyloTree project, which suggested that on average five germline mutations per sample were missed in the SGS analysis [82]. The error rate per base is higher in SGS data than in Sanger sequencing, but this can be compensated for by increasing the sequencing depth per base as demonstrated by a recent study where mtDNA was sequenced from 109 individuals and five discrepancies were found between Sanger sequencing and SGS data [63]. All of the discrepancies were due to problems with the base-calling software for Sanger sequencing [63]. Additional quality filtering of the SGS data can also be performed.

Although standard STR typing provides sufficient discrimination power for most applications, the use of large-scale sequencing provides multiple improvements to forensic analyses. For example, sequencing can identify SNPs within and around STRs, which can increase power in kinship analysis. An interesting option for the future would be to replace the STRs with more informative markers derived from large-scale sequencing. The major problem with such a replacement would be that the existing forensic databases are based on STR profiles, and in many cases forensic samples have not been stored for reanalysis. Unfortunately, the current SGS technologies are relatively poor at resolving STRs, and thus replacement of STR genotyping with SGS would require an immense amount of sequencing work to update the databases as well as collection of redundant genotype information during a transition phase. The benefits of analyzing degraded samples present in small quantities, simplified laboratory procedures, larger amount of data and accurate variant calling using SGS speak in favor of replacement of STRs with SGS in forensic analyses.

In a recent study, two Y chromosomes separated by 13 generations were sequenced using SGS technology [83,84]. Approximately one mutation per generation was identified, suggesting that every individual Y chromosome can be discriminated by sequencing. This holds promise for use in forensic applications since the Y-STRs used to identify male criminals in mixed samples, despite recent advances in identifying a set of rapidly evolving markers [85], have limited resolution in distinguishing between closely related men.

In recent years, genome-wide association studies have identified SNPs that can help predict ethnic background and appearance traits. This has a great interest for forensics, since such information can help identifying a criminal without the need of a reference sample. Several phenotypic traits, including eye, hair and skin color, can be relatively accurately (80% to 90%) predicted, while predicting body height is currently less accurate [86]. Also in this case, whole genome sequencing can improve resolution, by providing information on markers that are not present on genotyping chips.

The systems for single molecule sequencing with long read lengths that are now under development may turn out to be particularly advantageous for forensic genetics. The long read lengths would allow direct determination of mitochondrial haplogroups when several variants are present in the same read. Single molecule sequencing will also facilitate identification of multiple donors in a mixed sample. Another attractive possibility for handling of contaminated samples is to enrich for human or mtDNA material by target capture, similar to how Neanderthal DNA was enriched from contaminated samples using primer extension capture [74] and array-based capture [87].

In comparison with human samples, where a limited set of predesigned DNA markers is usually enough for forensic analysis, microbial samples provide an additional challenge since the markers are unknown and specific for each case. Microbial forensics has previously benefited from Sanger sequencing, for example for determination of the origin of the bacteria used in the well known 2001 anthrax attack in the USA [88,89]. To resolve this case, thousands of colonies were inspected manually, and a number of colonies with slightly different morphology were selected for whole genome sequencing [88,89]. Based on the identified polymorphisms, four tests were designed and used to screen more than 1,000 isolates from different laboratories. The cost of each anthrax genome in 2001 was estimated to US$140,000 [90]. In comparison, four *Bacillus anthracis *strains were recently sequenced on the SOLiD system with a reagent cost per genome of less than US$1,000 [91]. According to the current specifications of Illumina's HiSeq2000 instrument, approximately 300 × coverage of 384 *B. anthracis *isolates can be obtained from a single run at a reagent cost of less than US$50 per genome.

## Conclusions

The impressive advances of sequencing technologies in recent years have enabled a range of new applications, including population genetics based on the complete genomic sequences of a large number of individuals and sequencing of the complete genomes of highly contaminated samples of ancient DNA from humans and other species. The technical development of current SGS systems shows no sign of halting, with major upgrades from both the Illumina and SOLiD platforms in 2011. In forensic genetics, SGS is not yet widely employed, although a number of studies have highlighted emerging possibilities. With further decreased sequencing cost and miniaturized equipment, it will become possible for in principle any experimental research group to perform whole genome sequencing of both large and small genomes on a bench-top DNA sequencer. While the resources for data storage and analysis will initially be limiting, new simplified solutions for data storage and analysis are also underway. Before long, DNA sequencing is likely to become an easily accessible routine method, like the PCR technique is today.

## Competing interests

AK is currently employed by F. Hoffman-La Roche Ltd.

## Authors' contributions

ECB, AK and ACS wrote the manuscript. ECB performed graphical work. All authors read and approved the final manuscript.
